# Exposure to heavy metals and trace elements among pregnant women with twins: levels and association with twin growth discordance

**DOI:** 10.3389/fpubh.2024.1203381

**Published:** 2024-02-20

**Authors:** Lu Chen, Wei Zhao, Li Zhao, Qiongxin Liang, Jun Tang, Weixiao Zhou, Yanhua Zhang, Hong Wen

**Affiliations:** ^1^Department of Obstetrics, Women's Hospital, Zhejiang University School of Medicine, Hangzhou, China; ^2^Zhejiang Provincial Center for Disease Control and Prevention, Hangzhou, China

**Keywords:** thallium, heavy metals, trace elements, prenatal exposure, twin growth discordance

## Abstract

**Background:**

Twin growth discordance is one of the leading causes of perinatal mortality in twin pregnancies. Whether prenatal exposure to heavy metals and trace elements is associated with twin growth discordance has not been studied yet.

**Objective:**

To evaluate the prenatal level of heavy metals and trace elements in twin pregnancy and its relationship with twin growth discordance.

**Methods:**

This study involving 60 twin pairs and their mothers was conducted in Zhejiang Province, China, in 2020–2021. The concentration of heavy metals and trace elements in maternal blood, umbilical cord, and placenta were collected at delivery and measured by inductively coupled plasma tandem mass spectrometer. The association of prenatal level with twin growth discordance was evaluated using conditional logistic regression.

**Results:**

High levels of heavy metal elements (thallium in maternal blood and umbilical cord blood of larger twins, vanadium in the placenta of larger twins) and trace elements (iodine in the placenta of larger twins) during pregnancy, as well as low levels of heavy metal elements (strontium in the umbilical cord blood of larger twins, strontium and chromium in the umbilical cord blood of smaller twins, strontium in the placenta of larger twins, molybdenum and lead in the placenta of smaller twins and difference of molybdenum in the placenta of twins), are associated with intertwin birthweight discordance. Univariate regression analyses showed a significant effect of gestational age at delivery and eleven trace element data on intertwin birthweight discordance. Multivariable logistic regression analysis with transformed variables as dichotomous risk factors combined with baseline demographic characteristics showed Tl in maternal blood as an independent risk factor. The model constructed by combining Tl in maternal blood (OR = 54.833, 95% CI, 3.839–83.156) with the gestational week (OR = 0.618, 95% CI, 0.463–0.824) had good predictive power for intertwin birthweight discordance (AUC = 0.871). The sensitivity analysis results indicate that the effect of maternal blood thallium on intertwin birthweight discordance is stable and reliable.

**Conclusion:**

To our knowledge, ours is the first case–control study to investigate the association between elevated maternal thallium levels before delivery and twin growth discordance.

## 1 Introduction

A prevalent problem in twin pregnancies is twin growth discordance, which is typically characterized as an intertwin birth weight difference of at least 20% (1). It can affect up to 10–28.8% of twin gestations, and the incidence varies considerably between populations in different regions (1–4). Compared with growth-concordant twins, discordant twins lead to an increased incidence of perinatal mortality and morbidity (1, 5, 6), as well as delayed physical and neurological development in childhood, and have a sustained impact on health in adulthood (2, 7). Therefore, the identification of risk factors for twin growth discordance and reducing its incidence through early intervention can improve the quality of twin pregnancy care and have far-reaching societal implications.

The birth weight of twin pregnancies reflects, to some extent, the intrauterine environment, nutrition, and metabolic status of the fetus. Twin growth discordance has been associated with multiple risk factors, including advanced maternal age and parity, smoking, excess gestational weight gain, the use of assisted reproductive technology (ART), and pregnancy complications such as anemia, hypertensive disorders, and diabetes (8–10). Prenatal exposure to environmental pollutants is also believed to play a substantial influence in addition to those established contributory factors.

Exposure to environmental pollutants during pregnancy including ambient air pollutants and heavy metals was a risk factor for low birth weight (11). Two recent human epidemiological studies investigated the effects of environmental pollutants and atmospheric particles on fetal growth in twin pregnancies. One study reported that respirable particulate air pollutants (PM_10_) exposure in late gestation was associated with a significantly larger within-pair birth weight difference (12). Another study found that fine particulate matter air pollution (PM_2.5_) air pollution exposure in late gestation was a risk factor for twin growth discordance (13). However, no epidemiological study has investigated the association between prenatal exposure to metal and trace elements and the risk of twin growth discordance.

Heavy metal contamination and trace element deficiency are important public health issues in China (14, 15). High exposure to heavy metals or trace element deficiencies during pregnancy may be associated with the risk of many perinatal and fetal complications. Several studies have shown that exposure to certain heavy metals such as cadmium (16–19), nickel (20), manganese (21, 22), antimony (23), arsenic (24, 25) and lead (26–28) during pregnancy may inhibit fetal growth, thereby increasing the risk of low birth weight and small for gestational age infants. In addition, trace element deficiencies such as iron (29) and iodine (29) are also associated with intrauterine growth restriction in singleton pregnancies.

In this study, we evaluated the prenatal level of heavy metals and trace elements in twin pregnancy and investigated whether these element levels was associated with twin growth discordance in a twin birth cohort conducted in Hangzhou, China. Further, we also sought to identify the major elements which were responsible for the increased risk of twin growth discordance.

## 2 Materials and methods

### 2.1 Study design and participants

Twin pregnant women, who came for their first examination in the first trimester were recruited between January and December 2021 at the Women’s Hospital, Zhejiang University School of Medicine. The inclusion criteria included: (a) continuous residence in Hangzhou for ≥2 years with an expectation to reside continually for the foreseeable future, (b) subsequently conducting regular antenatal visits until delivery, (c) twin pregnancies born with 2 liveborn twins and delivered after 28 weeks of gestation, (d) no history of occupational exposure, and (e) no relevant polluting businesses in the residential area. The exclusion criteria included: (a) intrauterine treatment or fetal reduction during pregnancy, (b) known history of immunological, hypertension, diabetes, liver diseases, thyroid disorders, iron deficiency anemia, and kidney diseases before pregnancy, (c) smoking and drinking alcohol, (d) monochorionic monoamniotic twin or complications specific to monochorionic twins such as twin-to-twin transfusion syndrome and twin anemia-polycythemia sequence (TAPS), et al., (e) fetal aneuploidy and any structural or congenital anomaly, and (f) foreign pregnant women. All participants provided informed consent for participation in the study after receiving a detailed explanation of the study. The research protocol was approved by the Ethics Committee of Women’s Hospital, Zhejiang University School of Medicine (IRB-20210007-R).

### 2.2 Data collection and outcome definition

The face-to-face interviews were conducted by a trained interviewer in the hospitals with the participants after delivery using a standardized questionnaire to collect general information. The interview collected a variety of information, including socioeconomic data (e.g., maternal age, education, occupation, household income, and self-reported weight before pregnancy) and lifestyle factors during pregnancy (e.g., nutrient supplements and medications). Gestational age was estimated using the first-trimester ultrasonography and by measuring crown-rump length. The babies’ weight was measured within 10 min after delivery by experienced obstetric nurses using standardized procedures, and the information of the newborns’ birth date, gender, and the gestational week was also recorded. In twin pregnancies, the fetus with the larger birth weight was defined as larger twin and the fetus with the smaller birth weight was defined as smaller twin. The inter-twin birthweight discordance (IBWD) was calculated using the formula (A-B) × 100/A, where A is the birthweight of the larger twin and B is the birthweight of the smaller twin. Twin growth discordance was defined as IBWD ≥20%.

### 2.3 Exposure assessment

#### 2.3.1 Sample collection

The fasting peripheral venous maternal blood (5 mL) is collected within 24 h before delivery, and venous umbilical cord blood (2 mL) of both fetuses was collected at the time of delivery. The blood samples were obtained by venipuncture and collected in a Vacutainer tube (BD, Oakville, Ontario, Canada). The blood was centrifuged at 3000 rpm for 10 min. The blood samples were stored at −80°C until laboratory examination. All samples were processed within 15 min after collection. The placenta is divided into 8 parts at the center of the umbilical cord insertion on a sterile operating table, and three of the symmetrical portions (full thickness) were collected. After removing the decidua, amniotic membrane, and blood vessels visible to the naked eye, the placenta was rinsed in triple-steamed water and then squeezed to remove blood and water. And then the placenta was freeze-dried and ground to a powder before the elemental determination was carried out.

#### 2.3.2 Laboratory methods

All the collected blood and placenta samples were analyzed for heavy metals of vanadium (V), chromium (Cr), manganese (Mn), cobalt (Co), nickel (Ni), copper (Cu), zinc (Zn), strontium (Sr), molybdenum (Mo), cadmium (Cd), stannum (Sn), stibium (Sb), thallium (Tl), lead (Pb) and trace nutrient elements of arsenic (As), ferrum (Fe), iodine (I) and selenium (Se) using an inductively coupled plasma tandem mass spectrometer (ICP-MS). Briefly, blood and placental samples were digested into clear solution using a microwave digestion instrument (Ethos 1, Advanced Microwave Digestion System, Milestone, Sorisole, Italy) and then the sample solution was tested by ICP-MS system (iCAP Q, Thermo Fisher Scientific). The internal standards and quality control for multielement analysis were described in detail previously (30). The R-Squared values were greater than 0.999. The limit of detection (LOD) of the method for V, Cr, Mn, Co, Ni, Cu, Zn, Sr., Mo, Cd, Sn, Sb, Tl, Pb, Fe, As, Se, and I in placenta sample were, respectively, 0.040 ug/kg, 0.080 ug/kg, 0.100 ug/kg, 0.020 ug/kg, 0.260 ug/kg, 2.980 ug/kg, 0.714 ug/kg, 0.200 ug/kg, 0.080 ug/kg, 0.020 ug/kg, 0.220 ug/kg, 0.040 ug/kg, 0.020 ug/kg, 0.040 ug/kg, 0.426 ug/kg, 0.060 ug/kg, 0.020 ug/kg, and 1.660 ug/kg. The LOD of the method for V, Cr, Mn, Co, Ni, Cu, Zn, Sr., Mo, Cd, Sn, Sb, Tl, Pb, Fe, As, Se, and I in blood and cord blood sample were, respectively, 0.035 ug/ L, 0.069 ug/L, 0.111 ug/L, 0.021 ug/L, 0.186 ug/L, 0.337 ug/L, 0.700ug/L, 0.072 ug/L, 0.039 ug/L, 0.072 ug/L, 0.123 ug/L, 0.028 ug/L, 0.030 ug/L, 0.104 ug/L, 0.412ug/L, 0.035 ug/L, 0.110 ug/L, and 0.060 ug/L.

### 2.4 Statistical analysis

For each couple of twins, the outcome was defined as 0 for IBWD<20% and 1 otherwise, covariates investigated were maternal age, delivery gestational age, birth weight and gender of both twins, chorionicity, pregnancy complication and drug for thyroid disease; exposures were maternal heavy metals in blood, heavy metals measured in umbilical cord blood of larger and smaller twin, difference between heavy metals measured in umbilical cord blood of larger vs. smaller twin (same for placenta).

Data were analyzed using SPSS version 25.0 (SPSS Inc., Chicago, IL, United States). Descriptive statistics are presented as mean ± SD for normally distributed continuous variables and median (interquartile range) for non-normally distributed continuous variables. Normality was examined using the Shapiro–Wilk test. Categorical variables are presented as numbers (%). Comparison tests were performed using a *t*-test for normally distributed continuous variables, a Mann–Whitney U test for non-normally distributed continuous variables as well as a chi-square test for categorical variables. Then, Univariate logistic regression analysis was performed to identify risk factors, and statistically significant trace elements were analyzed using ROC curves, and then were categorically transformed using the maximum point of the Jorden index as the cutoff value. Subsequently, a multivariable logistic regression analysis was performed with these transformed variables as risk factors combined with baseline demographic characteristics to observe multifactorial and composite prediction results.

Additionally, we did three sensitivity analyses to identify the association between trace elements and intertwin birthweight discordance: (1) Continuous trace element in logistic regression instead of dummy; (2) Define the outcome as IBWD> = 30% instead of 20%; and (3) Use a linear outcome (and linear regression) instead of a dummy indicator. Statistical significance was defined as a two-sided *p*-value < 0.05 for all analyses.

## 3 Result

### 3.1 Subject characteristics

**Figure 1:**
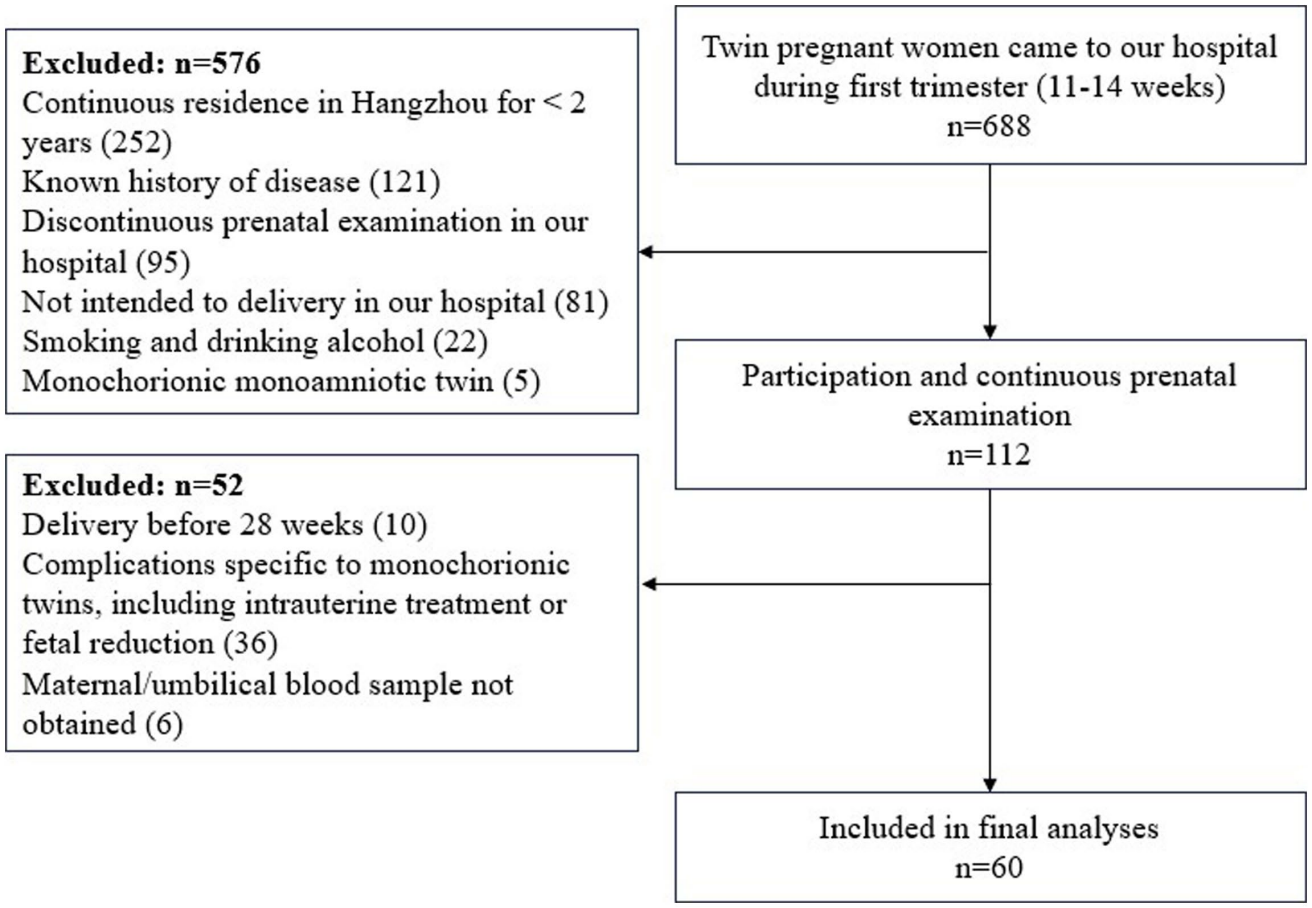
Selection of study population.

During the study period, 688 twin pregnant women came to our hospital during first trimester (11–14 weeks). Exclusion criteria was applied to 576 subjects (i.e., residence in Hangzhou for <2 years, *n* = 252; known history of disease, *n* = 121; discontinuous prenatal examination in our hospital, *n* = 95; not intended to delivery in our hospital, *n* = 81; Smoking and drinking alcohol, *n* = 22; monochorionic monoamniotic twin, *n* = 5). Ultimately, 112 subjects participated and examined continuously in our hospital. On the other hand, subjects were excluded if they delivered before 28 weeks (*n* = 10), or if they had complication specific to monochorionic twins, including intrauterine treatment or fetal reduction (*n* = 36), or maternal/umbilical blood sample not obtained (*n* = 6). Finally, a total number of 60 women and their twin newborns were included in the analyses (Figure 1).

The general characteristics of all participants are shown in Table 1. Among the 60 participating pregnant women, 35% (21) were IBWD (intertwin birthweight discordance) ≥20, and 65% (39) were IBWD<20%. They averaged 29.58 (SD = 3.92) years old and did not have the habit of cigarette smoking or alcohol drinking during this pregnancy. The pregnancy complication rates were 68.3 and 56.7% for thyroid medication. Of the 60 twin pairs, there were 25 (41.7%) dizygotic twins and 35 (58.3%) monozygotic twins. There were no significant differences in maternal age, neonatal gender, twin chorionicity, rate of pregnancy complication (gestational diabetes mellitus, hypertensive disorder complicating pregnancy), and thyroid medication between the two groups. The mean GA at delivery was 35 weeks, of which the mean gestational age at delivery was 34 weeks in the IBWD≥20% group and 36 weeks in the IBWD<20% group, and there was a statistically significant difference between the two groups, *p* = 0.002. The larger twins had a mean birthweight of 2,380 g, of which the mean birth weight of larger twins was 2,180 g in the IBWD≥20% group and 2,490 g in the IBWD<20% group and there was a statistically significant difference between the two groups, *p* = 0.001. The mean birth weight of smaller twins was 2157.50 g, of which the mean birth weight of smaller twins was 1,500 g in the IBWD≥20% group and 2,320 g in the IBWD<20% group, and there was a statistically significant difference between the two groups, *p* < 0.001.

**Table 1:** Comparison of the population and clinical characteristics between the IBWD≥20% and IBWD<20% groups.

	Total (*n* = 60)	IBWD≥20% (*n* = 21)	IBWD<20% (*n* = 39)	*p*
Age (years, mean ± sd)	29.58 ± 3.92	29.10 ± 3.69	29.85 ± 4.06	0.483
GA at delivery (weeks, median and IQR)	35 (2)	34 (4)	36 (2)	0.002
BW of larger twins (g, median and IQR)	2,380 (473)	2,180 (550)	2,490 (360)	0.001
BW of smaller twins (g, median and IQR)	2157.50 (858)	1,500 (700)	2,320 (310)	<0.001
Neonatal gender (*N* and %)				0.179
MM	31 (51.7)	7 (33.3)	24 (61.5)	
FF	18 (30.0)	9 (42.9)	9 (23.1)	
MF	7 (11.7)	3 (14.3)	4 (10.3)	
FM	4 (6.6)	2 (9.5)	2 (5.1)	
Chorionic (*N* and %)				0.416
DC	25 (41.7)	7 (33.3)	18 (46.2)	
MC	35 (58.3)	14 (66.7)	21 (53.8)	
Pregnancy complication (*N* and %)				0.154
Yes	41 (68.3)	17 (81.0)	24 (61.5)	
No	19 (31.7)	4 (19.0)	15 (38.5)	
Drugs for TD (*N* and %)				0.109
Yes	34 (56.7)	15 (71.4)	19 (48.7)	
No	26 (43.3)	6 (28.6)	20 (51.3)	

IQR, interquartile range; N, number; IBWD, intertwin birthweight discordance; GA, gestational age; BW, birth weight; MM, male–male; FF, female–female; MF, male–female; FM, female–male; DC, dichorionic; MC, monochorionic; TD, thyroid disease.

### 3.2 Exposure levels

#### 3.2.1 Heavy metals and trace elements in maternal blood

The median level of thallium in maternal blood was 26.085 ng/L, of which the median level of thallium in maternal blood was 42.214 ng/L in the IBWD≥20% group and 21.440 ng/L in the IBWD<20% group, and there was a statistically significant difference between the two groups, *p* = 0.002 (Table 2). For the remaining 17 elements, there was no significant difference in maternal blood levels between the two groups (Supplementary Table S1).

**Table 2:** Quantitative levels of trace elements grouped by intertwin birthweight discordance.

Element/Units	Total (*n* = 60)			IBWD≥20% (*n* = 21)	IBWD<20% (*n* = 39)	t/Z	*p*
	% of detection	Mean ± SD	Median (IQR)	Median (IQR)	Median (IQR)		
M_Tl/ng/L	84.62	107.197 ± 244.239	26.085 (20.528)	42.214 (750.965)	21.440 (13.737)	3.054	0.002
UL_Sr/μg/L	100	19.519 ± 6.230	20.244 (5.837)	16.768 (10.786)	21.141 (5.874)	2.599	0.013
UL_Tl//ng/L	47.73	85.511 ± 226.776	14.372 (8.758)	23.804 (759.842)	13.455 (6.451)	3.150	0.002
US_Cr/μg/L	100	0.686 ± 0.660	0.491 (0.366)	0.393 (0.185)	0.578 (0.336)	−2.393	0.017
US_Sr/μg/L	100	19.622 ± 6.323	20.134 (7.332)	16.532 (12.687)	21.384 (6.990)	2.157	0.048
PL_I/μg/kg	100	115.897 ± 216.627	54.227 (93.957)	80.449 (72.726)	35.491 (74.444)	2.474	0.013
PL_Sr/μg/kg	100	203.153 ± 322.026	92.569 (127.169)	72.681 (57.112)	108.566 (125.259)	−2.602	0.009
PL_V/μg/kg	93.22	0.591 ± 0.635	0.341 (0.715)	0.846 (1.256)	0.290 (0.430)	2.618	0.009
PS_Mo/μg/kg	100	11.603 ± 5.119	10.652 (6.019)	8.783 (4.932)	11.036 (7.254)	−3.198	0.001
PS_Pb/μg/kg	100	12.937 ± 8.437	10.611 (8.022)	8.093 (4.153)	12.162 (9.104)	−2.319	0.020
PD_Mo/μg/kg	100	1.044 ± 8.260	0.317 (5.771)	1.149 (7.016)	−0.440 (6.512)	2.485	0.013

IBWD, intertwin birthweight discordance; M, maternal blood; UL, umbilical cord blood of larger twin; US, umbilical cord blood of smaller twin; PL, placenta of larger twin; PS, placenta of smaller twin; PD, differences of trace elements in placenta of twins (PL-PS); Tl, thallium; Sr, strontium; Cr, chromium; V, vanadium; I, iodine; Mo, molybdenum; Pb, lead.

#### 3.2.2 Trace elements in umbilical cord blood of both twins

The median umbilical cord blood levels of Tl of larger twins were 14.372 ng/L, of which the umbilical cord blood levels of Tl of larger twins was 23.804 ng/L in the IBWD≥20% group and 13.455 ng/L in the IBWD<20% group, and there was a statistically significant difference between the two groups, *p* = 0.002. The median umbilical cord blood levels of Sr. of larger twins were 20.244ug/L, of which the umbilical cord blood levels of Sr. of larger twins were 16.768ug/L in the IBWD≥20% group and 21.141ug/L in the IBWD<20% group, and there was a statistically significant difference between the two groups, *p* = 0.013 (Table 2). For the remaining trace elements, there was no significant difference in the umbilical cord blood levels of larger twins between the two groups (Supplementary Table S2).

The median umbilical cord blood levels of Cr of smaller twins were 0.491ug/L, of which the umbilical cord blood levels of Cr of smaller twins were 0.393ug/L in the IBWD≥20% group and 0.578ug/L in the IBWD<20% group, and there was a statistically significant difference between the two groups, *p* = 0.017. The median umbilical cord blood levels of Sr. of smaller twins were 20.134ug/L, of which the umbilical cord blood levels of Sr. of smaller twins were 16.532ug/L in the IBWD≥20% group and 21.384ug/L in the IBWD<20% group, and there was a statistically significant difference between the two groups, *p* = 0.048 (Table 2). For the remaining trace elements, there was no significant difference in the umbilical cord blood levels of smaller twins between the two groups (Supplementary Table S3). The differential levels of 18 trace elements in the umbilical cord blood of both twins were not significantly different between the two groups (Supplementary Table S4).

#### 3.2.3 Trace elements in the placenta of both twins

The median placenta levels of V of larger twins were 0.846 ug/kg in the IBWD≥20% group and 0.290 ug/kg in the IBWD<20% group, and there was a statistically significant difference between the two groups, *p* = 0.009. The median placenta levels of Sr. of larger twins were 92.569 ug/kg, of which the placenta levels of Sr. of larger twins were 72.681 ug/kg in the IBWD≥20% group and 108.566ug/kg in the IBWD<20% group and there was a statistically significant difference between the two groups, *p* = 0.009. The median placenta levels of I of larger twins was 54.227ug/kg, of which the placenta levels of I of larger twins were 80.449ug/kg in the IBWD≥20% group and 35.491 ug/kg in the IBWD<20% group, there was a statistically significant difference between the two groups, *p* = 0.013 (Table 2). For the remaining trace elements, there was no significant difference in the placenta levels of larger twins between the two groups (Supplementary Table S5).

The median placenta levels of Mo of smaller twins were 8.783ug/kg in the IBWD≥20% group and 11.036ug/kg in the IBWD<20% group, and there was a statistically significant difference between the two groups, *p* = 0.001. The placenta levels of Pb of smaller twins were 8.093ug/kg in the IBWD≥20% group and 12.162ug/kg in the IBWD<20% group, and there was a statistically significant difference between the two groups, *p* = 0.020 (Table 2). For the remaining trace elements, there was no significant difference in the placenta levels of smaller twins between the two groups (Supplementary Table S6).

The median differential levels of Mo in the placenta of both twins were 1.149 ug/kg in the IBWD≥20% group and − 0.440ug/kg in the IBWD<20% group, and there was a statistically significant difference between the two groups, *p* = 0.013 (Table 2). For the remaining trace elements, there was no significant difference in the differential levels in the placenta of both twins between the two groups (Supplementary Table S7).

### 3.3 Risk factors for intertwin birthweight discordance

**Figure 2:**
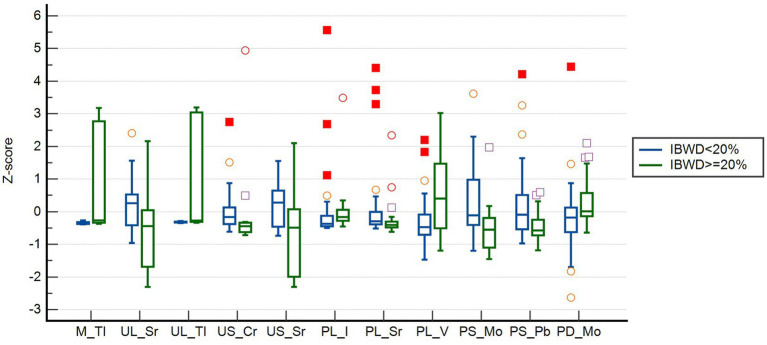
Normalized blood concentration distributions of 11 trace elements at different IBWD group. ○: represent an outlier if it is at least 1.5 interquartile ranges below the first quartile, or at least 1.5 interquartile ranges above the third quartile; □: represent an extreme if it is at least 3 interquartile ranges below the first quartile, or at least 3 interquartile ranges above the third quartile.

The subject operating characteristic curve (ROC) analysis showed that all trace elements that were significant in the univariate analysis had some predictive value for intertwin birthweight discordance. Among them, the maternal blood levels of Tl had the best predictive performance, with an AUC of 0.805. The maximum Jorden index point of 38 was used as the cutoff value, which had a sensitivity of 89.5% and a specificity of 59.0% (Table 3). The box plots of the distribution of these 11 trace elements among different groups showed that the distribution of these trace elements was not consistent among different groups, especially the Tl levels in maternal blood and in umbilical cord blood of larger twins, which were significantly higher in the IBMD≥20% group than in the IBWD<20% group (Figure 2).

**Table 3:** Accuracy for prediction of birthweight discordance using single trace elements.

Element/Units	Cutoff value	AUC	Specificity	Sensitivity
M_Tl/ng/L	38	0.805	0.590	0.895
UL_Sr/μg/L	19.858	0.736	0.641	0.800
UL_Tl/ng/L	21	0.799	0.667	0.846
US_Cr/μg/L	0.485	0.733	0.769	0.650
US_Sr/μg/L	20.285	0.733	0.633	0.846
PL_I/μg/kg	53	0.699	0.590	0.789
PL_Sr/μg/kg	78	0.709	0.700	0.786
PL_V/μg/kg	1	0.710	1.000	0.571
PS_Mo/μg/kg	11	0.761	0.462	0.947
PS_Pb/μg/kg	11	0.690	0.962	0.615
PD_Mo/μg/kg	−1	0.703	0.795	0.600

M, maternal blood; UL, umbilical cord blood of larger twin; US, umbilical cord blood of smaller twin; PL, placenta of larger twin; PS, placenta of smaller twin; PD, differences of trace elements in placenta of twins (PL-PS); Tl, thallium; Sr, strontium; Cr, chromium; V, vanadium; I, iodine; Mo, molybdenum; Pb, lead.

A univariate regression analysis of maternal age, gestational age at delivery, neonatal gender, chorionicity, pregnancy complications, thyroid medication use, and trace elements (after dichotomous conversion of cutoff values) with intertwin birthweight discordance showed a significant effect of gestational age at delivery and eleven trace element data on the outcome (Table 4).

**Table 4:** Univariate regression analysis with different variables.

Variables	OR (95%CI)	*p*
Age (years)	0.951 (0.827–1.093)	0.477
GA at delivery (weeks)	0.690 (0.543–0.878)	0.002
Neonatal gender		0.231
MM	Reference	
FF	0.292 (0.035–2.462)	0.258
MF	1.000 (0.115–8.730)	1.000
FM	0.750 (0.064–8.834)	0.819
Chorionic		0.339
DC	Reference	
MC	1.714 (0.568–5.172)	
Pregnancy complication	2.656 (0.749–9.420)	0.130
Drugs for TD	2.500 (0.799–7.821)	0.115
M_Tl /ng/L (≥38 vs. <38)	19.000 (2.141–168.594)	0.008
UL_Sr /μg/L (≥19.858 vs. <19.858)	0.143 (0.036–0.565)	0.006
UL_Tl /ng/L (≥21 vs. <21)	23.385 (2.664–205.250)	0.004
US_Cr /μg/L (≥0.485 vs. <0.485)	0.100 (0.020–0.489)	0.004
US_Sr/μg/L (≥20.285 vs. <20.285)	0.111 (0.023–0.541)	0.007
PL_I/μg/kg (≥53 vs. <53)	5.714 (1.724–18.944)	0.004
PL_Sr/μg/kg (≥78 vs. <78)	0.172 (0.054–0.549)	0.003
PL_V/μg/kg (≥1 vs. <1)	7.385 (1.697–32.139)	0.008
PS_Mo/μg/kg (≥11 vs. <11)	0.100 (0.020–0.489)	0.004
PS_Pb/μg/kg (≥11 vs. <11)	0.164 (0.046–0.578)	0.005
PD_Mo/μg/kg (≥ − 1 vs. < −1)	5.143 (1.300–20.338)	0.020

GA, gestational age; DC, dichorionic; MC, monochorionic; TD, thyroid disease; M, maternal blood; UL, umbilical cord blood of larger twin; US, umbilical cord blood of smaller twin; PL, placenta of larger twin; PS, placenta of smaller twin; PD, differences of trace elements in placenta of twins (PL-PS); Tl, thallium; Sr, strontium; Cr, chromium; V, vanadium; I, iodine; Mo, molybdenum; Pb, lead.

### 3.4 Predictive model for intertwin birthweight discordance

**Figure 3:**
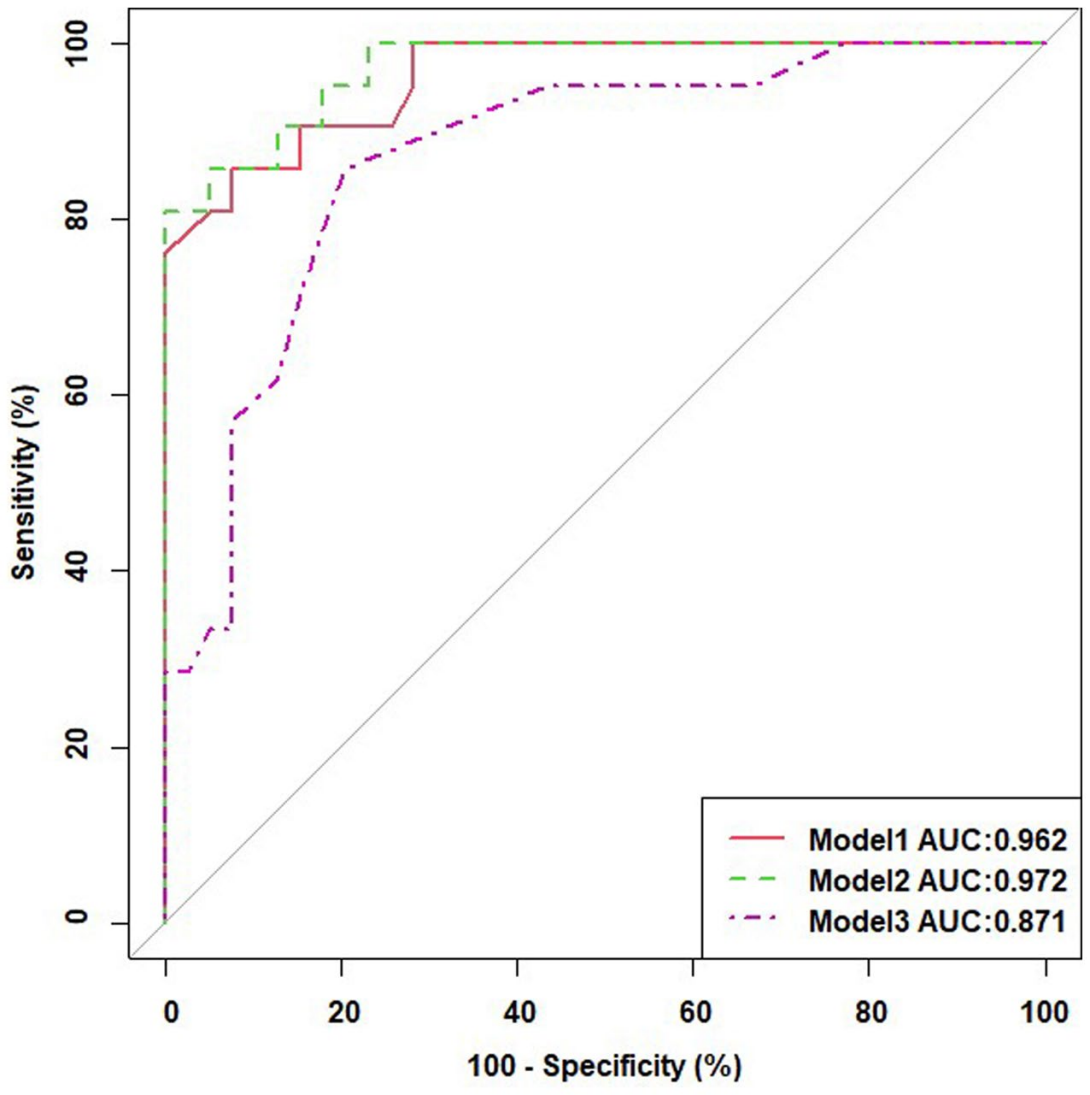
ROC curves for Model 1, Model 2 and Model 3.

Multivariable logistic regression analysis was performed with transformed variables as risk factors combined with baseline demographic characteristics to observe multifactorial and composite prediction results (Table 5). Model 1 was built with 11 features; the areas under the ROC curves (AUCs) for predicting intertwin birthweight discordance was 0.962. Model 2 was built with 12 features, including elements in Model 1 and gestational age at delivery, with AUCs of 0.972 (Figure 3). After ranking these features, it was found that these factors were no longer significant except for Tl in maternal blood which was still significant without the addition of gestational age at delivery. Gestational age at delivery and Tl in maternal blood were finally retained in Model3, and AUCs was 0.871, with the OR for gestational age at delivery was 0.618, 95% CI 0.463 to 0.824, the OR for Tl in maternal blood was 54.833, 95% CI 3.839 to 83.156 (Figure 3).

**Table 5:** Multivariable regression analysis with different variables.

Variables/Units	Model 1	Model 2	Model 3
	OR (95%CI)	OR (95%CI)	OR (95%CI)
GA at delivery (weeks)		0.458 (0.209, 1.005)	0.618 (0.463, 0.824)
M_Tl (≥38 vs. <38 ng/L)	>999.999 (<0.001, >999.999)	>999.999 (<0.001, >999.999)	54.833 (3.839, 783.156)
UL_Sr (≥19.858 vs. <19.858 μg/L)	0.041 (<0.001, 3.623)	0.083 (<0.001, 50.374)	
UL_Tl (≥21 vs. <21 ng/L)	1.076 (0.022, 52.012)	0.853 (0.003, 222.335)	
US_Cr (≥0.485 vs. <0.485 μg/L)	<0.001 (<0.001, >999.999)	<0.001 (<0.001, >999.999)	
US_Sr (≥20.285 vs. <20.285 μg/L)	2.740 (0.023, 319.898)	35.59 (0.032, >999.999)	
PL_I (≥53 vs. <53 μg/kg)	7.621 (0.657, 88.460)	7.659 (0.286, 205.160)	
PL_Sr (≥78 vs. <78 μg/kg)	0.362 (0.027, 4.828)	0.253 (0.015, 4.276)	
PL_V (≥1 vs. <1 μg/kg)	5.233 (0.135, 202.262)	9.893 (0.035, >999.999)	
PS_Mo (≥11 vs. <11 μg/kg)	0.016 (<0.001, 0.905)	0.126 (0.001, 14.843)	
PS_Pb (≥11 vs. <11 μg/kg)	2.283 (0.104, 50.043)	0.992 (0.038, 25.705)	
PD_Mo (≥ − 1 vs. < −1 μg/kg)	1.398 (0.107, 18.286)	0.303 (0.010, 8.768)	

GA, gestational age; M, maternal blood; UL, umbilical cord blood of larger twin; US, umbilical cord blood of smaller twin; PL, placenta of larger twin; PS, placenta of smaller twin; PD, differences of trace elements in placenta of twins (PL-PS); Tl, thallium; Sr, strontium; Cr, chromium; V, vanadium; I, iodine; Mo, molybdenum; Pb, lead.

To further evaluate the stability of the logistic regression results, we conducted sensitivity analysis for primary outcome (Table 6). Table 6 show that maternal blood thallium is still significant in the stepwise regression of Model S1. In Model S2, although maternal blood thallium is not significant, the OR value is greater than 1, demonstrating similarity with other results. In both models of Model S3, there was a significant positive correlation between maternal blood thallium and the results. The sensitivity analysis results indicate that the effect of maternal blood thallium on intertwin birthweight discordance is stable and reliable.

**Table 6:** Sensitivity analysis with different models.

	Model S1		Model S2	Model S3			
	OR (95%CI)^a^	OR (95%CI)^b^	OR (95%CI)^a^	Coefficient^a^	*p*^a^	Coefficient^b^	*p*^b^
Intercept				2.251	0.046	74.124	0.003
GA at delivery (weeks)	<0.001 (<0.001, 97.986)		0.218 (<0.001, >999.999)	−0.044	0.117	−1.735	0.006
M_Tl/ng/L	9.665 (0.465, 200.736)	1.126 (1.015, 1.249)	1.371 (0.213, 8.820)	0.008	0.029	0.302	0.002
UL_Sr/ug/L	0.001 (<0.001, 6.206)		0.14 (<0.001, >999.999)	−0.033	0.176		
UL_Tl//ng/L	0.152 (0.008, 2.949)		0.695 (0.101, 4.776)	−0.009	0.024	−0.289	0.003
US_Cr/ug/L	<0.001 (<0.001, >999.999)		<0.001 (<0.001, >999.999)	−0.096	0.230		
US_Sr/ug/L	>999.999 (0.079, >999.999)		2.983 (<0.001, >999.999)	0.016	0.498		
PL_I/μg/kg	1.139 (0.815, 1.591)		1.023 (0.806, 1.299)	0.0003	0.495		
PL_Sr/μg/kg	1.036 (0.991, 1.082)		0.999 (0.906, 1.101)	−0.000003	0.987		
PL_V/μg/kg	<0.001 (<0.001, >999.999)		>999.999 (<0.001, >999.999)	0.039	0.753		
PS_Mo/μg/kg	2.794 (0.131, 59.604)		1.019 (<0.001, >999.999)	−0.009	0.576		
PS_Pb/μg/kg	0.848 (0.073, 9.868)		0.707 (0.001, 359.436)	−0.004	0.721		
PD_Mo/μg/kg	8.685 (0.495, 152.424)		0.557 (<0.001, 395.595)	−0.001	0.931	0.301	0.045

Model S1: Continuous trace element in logistic regression instead of dummy. Model S2: Define the outcome as IBWD > =30% instead of 20%. Model S3: Use a linear outcome (and linear regression) instead of a dummy indicator. Continuous trace elements were used in all models.

a All variables enter the model.

b Stepwise method was used to select the variables, Model 2 had no variables entering the final model, so it is not shown.

GA, gestational age; UL, umbilical cord blood of larger twin; US, umbilical cord blood of smaller twin; PL, placenta of larger twin; PS, placenta of smaller twin; PD, differences of trace elements in placenta of twins (PL-PS).

## 4 Discussion

Numerous studies have investigated the effects of heavy metals or trace elements exposure on fetal growth in singletons. Although heterogeneity of the study population, study settings, and exposure assessment methods exist, most studies revealed that maternal exposure to heavy metals or trace elements deficiency during a specific period of pregnancy was associated with reduced fetal growth. In a population of twin pregnancies in Hangzhou, China, we applied the first inductively coupled plasma mass spectrometry system to detect metals and trace elements in umbilical cord blood, maternal blood, and placenta of twin fetuses by ICP-MS and investigated the effects of prenatal heavy metals or trace elements level on the fetal growth in twins and its key chemical constituents.

We demonstrated that higher levels of Tl in the maternal blood and umbilical cord blood of larger twins, V and I in the placenta of larger twins were significantly associated with increased intertwin birth weight discordance. Moreover, we observed a significantly increased risk of twin growth discordance in association with decreased levels of Sr. in the umbilical cord blood of both twins, Cr in the umbilical cord blood of smaller twins, Sr. in the placenta of larger twins, Mo and Pb in the placenta of smaller twins and difference of Mo in the placenta of twins. The associations of Tl in maternal blood with twin growth discordance were robust even after performing multiple logistic regression analyses of these transformed variables as risk factors combined with baseline demographic characteristics. The sensitivity analysis results show that regardless of using thallium directly as a continuity indicator, defining the outcome as IBWD> = 30% instead of 20%, or directly using fetal birth weight as continuous variable, the effect of maternal blood thallium on intertwin birthweight discordance is stable. Our results indicate that elevated maternal thallium levels before delivery are associated with twin growth discordance.

In a population of twin pregnancies in Hangzhou, China, we investigated the effects of prenatal heavy metals and trace elements level and focused on the key constituents as risk factors for fetal growth in twins. The pregnant women in our study had lower levels of Tl in maternal blood (median of 0.03 μg/L) compared with the general populations in other cities of China, such as in the Ma’anshan [geometric mean, 0.04871 μg/L (31); geometric mean, 0.04911 μg/L (32)], and other four cities in China [median, 0.2 μg/L (33)]. Compared with pregnant women in previous studies, our study population also had lower levels of Tl in umbilical cord blood (median of 0.01 μg/L in the larger fetus, a median of 0.02 μg/L in the smaller fetus) than those reported in pregnant women from the Ma’anshan [geometric mean, 0.03866 μg/L (32); median, 0.384 (32)], and other four cities in China [median, 0.04 μg/g (33)]. However, differences between studies may be caused by regional and racial differences and the different modes of exposure. The thallium levels in the umbilical cord blood of both large and small fetuses in this study were lower than the maternal blood level, while thallium was barely detectable in the placenta. We observed that the higher maternal thallium level can be associated with the higher thallium level in cord blood, and this also indicates that, the placenta cannot prevent its transfer from mother to fetus and protect the fetus from its harmful effect even with a low thallium level in blood cells of the mother.

Numerous studies have investigated the effects of prenatal thallium level on fetal growth in singletons. Although heterogeneity of the study population, study settings, and exposure assessment methods exist, most studies revealed that maternal exposure to thallium during a specific period of pregnancy was associated with reduced fetal growth (34). A small prospective study by Hu et al. reported a negative correlation between maternal blood T1 concentration and birth weight in 81 pairs of Chinese mothers and infants (33). In a case–control study conducted in Hubei Province, Xia et al. demonstrated that higher maternal urinary Tl levels were significantly associated with increased risk of LBW, and stratified analyses showed slightly higher risk estimates for LBW associated with higher Tl levels for mothers <28 years old and for mothers with lower household income (35). A study examining the combined effects of prenatal exposures to environmental chemicals on birth weight showed that the co-exposure of thallium, PFOS, lead, cadmium, manganese, and mercury was associated with reduced birth weight in girls (36). Qi et al. investigate the effects of prenatal Tl exposures on early child growth and development aged 0–2 years in a prospective birth cohort study and found umbilical cord serum Tl levels tended to be reduced a child’s stature and weight in young girls (37). The aforementioned studies suggest that thallium exposure can affect fetal growth in singleton fetuses, increasing the risk of growth restriction and low birth weight in newborns, and that the effects may continue into childhood. Our study confirms that thallium level in twin fetuses increases the risk of twin growth discordance and shows a decrease in birthweight of both large and small fetuses, but an increase in weight discordance compared to the growth-concordant twin.

The biological mechanisms underlying the associations of thallium exposure with twin growth discordance are not clear. Some placental factors, including lower placental weight and abnormal placental function, are linked to twin growth discordance. Studies have shown that thallium exposure could affect fetal development through a variety of mechanisms, including disturbing mitochondrial function in the placenta and fetal tissue (38, 39), triggering oxidative stress through increasing lipid oxidation (40), decreasing thyroid hormone levels (41), and inhibiting enzymes with active sites containing cysteine residues (42). A population study found that for every 25% increase in cord blood thallium levels in a singleton pregnancy, the mitochondrial mtDNA content of the placenta decreased by 4.88% (43). A previous study found the placental mtDNA fold changes between the small and large twins are different in monochorionic twin pregnancies with selective intrauterine growth restriction (sIUGR). Therefore, we speculate that the effects of thallium level on fetal growth in twins could be related to placental mitochondrial DNA and abnormal placental function, but more studies are needed to investigate the underlying mechanisms.

Our study adds to the literature on thallium level and adverse fetal outcomes in twins, and the best of our knowledge is the first investigation to report significant associations between prenatal level of certain heavy metals and twin growth discordance. Thallium exposure during pregnancy is inevitable due to the increasing release of thallium into the environment, where the consumption of contaminated food and drinking water is the main source of exposure. Multiple factors during pregnancy including pre-pregnancy BMI (44), maternal age (45), dietary habits (46), and intake of multivitamin supplements (47) can all influence thallium exposure levels. Considering twin growth discordance is a major cause of infant mortality and morbidity in twins and has lifetime health issues in multiple organs or systems, the increased odds ratio for twin growth discordance brought by thallium exposure may have significant clinical and public health importance.

However, our study is subject to serval limitations. Firstly, the present study only measured heavy metal and trace element concentrations in biological samples from 60 mothers and infants, and the findings need to be further supported and validated by evidence from a large cohort study. Secondly, different trimesters of thallium exposure should be considered to identify sensitive periods to thallium exposure and undertake interventions. A prospective study of Tl concentrations at multiple points may help to evaluate whether there is a critical exposure window of Tl on fetal development. Thirdly, numerous factors that could have increased the chance of twin growth discrepancies, such as the mother’s socioeconomic level, nutritional status, passive smoking, and physical health during pregnancy, were not available, and as a result, were not taken into account in this study. Finally, zygosity and chorionicity are not consistent. In this study, chorionicity in twin pregnancies was not a risk factor for discordant twins. Since there was no data on twin zygosity, we did not account for it in our regression models.

## 5 Conclusion

Our findings indicate that elevated maternal thallium levels before delivery are associated with twin growth discordance. These imply that twin pregnant women should avoid exposure to heavy metals, especially Tl. Further researches are necessary to confirm our findings, explore the potential biological mechanisms and develop strategies for reducing IBWD related to developmental exposure to environmental pollutants, including Tl.

## Data availability statement

The datasets presented in this study can be found in online repositories. The names of the repository/repositories and accession number(s) can be found in the article/Supplementary material.

## Ethics statement

The studies involving humans were approved by Ethics Committee of Women’s Hospital, Zhejiang University School of Medicine. The studies were conducted in accordance with the local legislation and institutional requirements. The participants provided their written informed consent to participate in this study.

## Author contributions

LC: conceptualization and writing – original draft preparation. WZha: resources, data curation, and supervision. LZ and QL: resources, data curation, and software. JT: investigation, formal analysis, and validation. WZho and YZ: visualization and investigation. HW: methodology and writing – reviewing and editing. All authors contributed to the article and approved the submitted version.
