# Supplementary material for: Exposure to heavy metals and trace elements among pregnant women with twins: levels and association with twin growth discordance
Source: Front Public Health. 2024 Feb 20;12:1203381. doi: 10.3389/fpubh.2024.1203381 (PMC10912306; doi:10.3389/fpubh.2024.1203381)
Supplement: Supplementary file 1 [file Data_Sheet_1.docx]

**Supplementary Material**

**Table 1**

Quantitative levels of trace elements in maternal blood grouped by intertwin birthweight discordance

| Element/Units | Total  (n=60) | | | IBWD³20%  (n=21) | IBWD<20%  (n=39) | *P* |
| --- | --- | --- | --- | --- | --- | --- |
|  | % of detection | Mean±SD | Median  (IQR) | Median | Median |  |
|  |  |  |  | (IQR) | (IQR) |  |
| M_V/ug/L | 87.18 | 0.108±0.075 | 0.113(0.104) | 0.091(0.074) | 0.117(0.107) | n.s. |
| M_Cr/ug/L | 100 | 0.671±0.379 | 0.589(0.458) | 0.652(0.454) | 0.588(0.416) | n.s. |
| M_Mn/ug/L | 100 | 24.935±7.722 | 24.701(12.709) | 24.273(7.059) | 24.838(13.523) | n.s. |
| M_Co/ug/L | 100 | 0.559±0.185 | 0.509(0.220) | 0.438(0.097) | 0.567(0.270) | n.s. |
| M_Ni/ug/L | 100 | 1.665±0.816 | 1.346(0.621) | 1.413(2.239) | 1.307(0.465) | n.s. |
| M_Cu/ug/L | 100 | 1727.189±244.895 | 1700.860(389.678) | 1680.66(294.126) | 1705.986(399.532) | n.s. |
| M_Zn/ug/L | 100 | 5932.562±1143.712 | 6092.237(1530.178) | 6269.711(1399.086) | 5959.457(1283.472) | n.s. |
| M_As/ug/L | 100 | 1.982±1.707 | 1.330(1.376) | 1.161(0.981) | 1.548(1.269) | n.s. |
| M_Se/ug/L | 100 | 118.099±33.550 | 112.575(33.573) | 103.937(22.586) | 114.501(52.000) | n.s. |
| M_Sr/ug/L | 100 | 27.155±9.894 | 28.183(14.048) | 22.148(17.117) | 29.183(9.458) | n.s. |
| M_Mo/ug/L | 100 | 0.517±0.222 | 0.494(0.255) | 0.521(0.220) | 0.448(0.255) | n.s. |
| M_Cd/ug/L | 100 | 0.588±0.527 | 0.411(0.488) | 0.395(0.130) | 0.471(0.644) | n.s. |
| M_Sn/ug/L | 100 | 0.730±2.492 | 0.258(0.141) | 0.282(0.342) | 0.256(0.081) | n.s. |
| M_Sb/ug/L | 100 | 8.077±13.368 | 6.188(2.138) | 5.018(1.794) | 6.592(2.104) | n.s. |
| M_I/ug/L | 100 | 243.496±383.347 | 77.771(157.224) | 81.833(52.295) | 72.581(157.224) | n.s. |
| M_Tl/ug/L | 84.62 | 0.107±0.244 | 0.026(0.021) | 0.042(0.751) | 0.021(0.014) | 0.002 |
| M_Pb/ug/L | 100 | 12.062±6.311 | 9.818(5.616) | 13.021(9.283) | 9.187(3.895) | n.s. |
| M_Fe/mg/L | 100 | 311.014±49.346 | 312.536(49.904) | 312.667(67.206) | 308.246(44.145) | n.s. |

Abbreviations: IBWD, intertwin birthweight discordance; M, maternal blood; V, vanadium; Cr, chromium; Mn, manganese; Co, cobalt; Ni, nickel; Cu, copper; Zn, zinc; As, arsenic; Se, selenium; Sr, strontium; Mo, molybdenum; Cd, cadmium; Sn, stannum; Sb, stibium; I, iodine; Tl, thallium; Pb, lead; Fe, ferrum

**Table 2**

Quantitative levels of trace elements in umbilical cord blood of larger twin grouped by intertwin birthweight discordance

| Element/Units | Total  (n=60) | | | IBWD≥20%  (n=21) | IBWD<20% (n=39) | *P* |
| --- | --- | --- | --- | --- | --- | --- |
|  | % of detection | Mean±SD | Median  (IQR) | Median | Median |  |
|  |  |  |  | (IQR) | (IQR) |  |
| UL_V/ug/L | 93.18 | 0.105±0.051 | 0.110(0.074) | 0.109(0.089) | 0.110(0.073) | n.s. |
| UL_Cr/ug/L | 100 | 1.120±3.177 | 0.424(0.338) | 0.339(0.345) | 0.513(0.301) | n.s. |
| UL_Mn/ug/L | 100 | 58.934±19.210 | 51.601(26.395) | 49.441(26.714) | 55.604(26.268) | n.s. |
| UL_Co/ug/L | 100 | 0.501±0.199 | 0.458(0.167) | 0.403(0.192) | 0.467(0.125) | n.s. |
| UL_Ni/ug/L | 100 | 1.481±0.891 | 1.170(0.275) | 1.318(2.69) | 1.160(0.143) | n.s. |
| UL_Cu/ug/L | 100 | 612.575±92.783 | 599.767(83.051) | 579.284(84.205) | 609.586(88.231) | n.s. |
| UL_Zn/ug/L | 100 | 1801.310±336.344 | 1741.639(306.4) | 1600.526(400.692) | 1794.970(264.229) | n.s. |
| UL_As/ug/L | 100 | 1.533±1.160 | 1.039(1.139) | 0.789(0.906) | 1.117(1.405) | n.s. |
| UL_Se/ug/L | 100 | 115.471±30.063 | 110.561(25.890) | 104.105(28.071) | 111.765(22.455) | n.s. |
| UL_Sr/ug/L | 100 | 19.519±6.230 | 20.244(5.837) | 16.768(10.786) | 21.141(5.874) | 0.013 |
| UL_Mo/ug/L | 100 | 0.439±0.161 | 0.393(0.176) | 0.378(0.334) | 0.399(0.156) | n.s. |
| UL_Cd/ug/L | 84.09 | 0.072±0.057 | 0.055(0.037) | 0.047(0.174) | 0.057(0.033) | n.s. |
| UL_Sn/ug/L | 100 | 0.298±0.363 | 0.215(0.073) | 0.205(0.357) | 0.217(0.071) | n.s. |
| UL_Sb/ug/L | 100 | 6.886±3.362 | 6.771(3.468) | 6.030(2.776) | 6.973(3.445) | n.s. |
| UL_I/ug/L | 100 | 77.500±55.979 | 49.645(59.868) | 64.604(79.399) | 48.390(54.659) | n.s. |
| UL_Tl/ug/L | 47.73 | 0.086±0.227 | 0.014(0.009) | 0.024(0.760) | 0.013(0.006) | 0.002 |
| UL_Pb/ug/L | 100 | 8.696±4.236 | 7.181(4.336) | 8.367(9.269) | 6.617(3.250) | n.s. |
| UL_Fe/mg/L | 100 | 381.972±50.550 | 390.007(61.311) | 388.733(55.290) | 390.571(64.162) | n.s. |

Abbreviations: UL, umbilical cord blood of larger twin; V, vanadium; Cr, chromium; Mn, manganese; Co, cobalt; Ni, nickel; Cu, copper; Zn, zinc; As, arsenic; Se, selenium; Sr, strontium; Mo, molybdenum; Cd, cadmium; Sn, stannum; Sb, stibium; I, iodine; Tl, thallium; Pb, lead; Fe, ferrum

**Table 3**

Quantitative levels of trace elements in umbilical cord blood of smaller twin grouped by intertwin birthweight discordance

| Element/Units | Total  (n=60) | | | IBWD ≥20% (n=21) | IBWD<20% (n=39) | *P* |
| --- | --- | --- | --- | --- | --- | --- |
|  | % of detection | Mean±SD | Median  (IQR) | Median  (IQR) | Median  (IQR) |  |
| US_V/ug/L | 95.35 | 0.107±0.061 | 0.104(0.077) | 0.082(0.098) | 0.104(0.067) | n.s. |
| US_Cr/ug/L | 100 | 0.686±0.660 | 0.491(0.366) | 0.393(0.185) | 0.578(0.336) | 0.017 |
| US_Mn/ug/L | 100 | 65.919±25.225 | 59.607(37.501) | 65.334(47.427) | 59.430(33.546) | n.s. |
| US_Co/ug/L | 100 | 0.513±0.165 | 0.508(0.206) | 0.465(0.287) | 0.510(0.173) | n.s. |
| US_Ni/ug/L | 100 | 1.898±1.872 | 1.271(0.609) | 1.396(2.39) | 1.243(0.393) | n.s. |
| US_Cu/ug/L | 100 | 653.308±144.843 | 641.701(100.801) | 604.696(177.144) | 643.893(85.727) | n.s. |
| US_Zn/ug/L | 100 | 1800.152±314.323 | 1792.028(311.434) | 1601.706(338.608) | 1801.940(269.224) | n.s. |
| US_As/ug/L | 100 | 1.631±1.246 | 1.316(0.969) | 0.824(0.839) | 1.375(1.290) | n.s. |
| US_Se/ug/L | 100 | 117.809±36.935 | 109.641(23.986) | 103.996(20.134) | 114.872(18.821) | n.s. |
| US_Sr/ug/L | 100 | 19.622±6.323 | 20.134(7.332) | 16.532(12.687) | 21.384(6.99) | 0.048 |
| US_Mo/ug/L | 100 | 0.419±0.223 | 0.349(0.129) | 0.379(0.300) | 0.345(0.090) | n.s. |
| US_Cd/ug/L | 79.07 | 0.074±0.055 | 0.056(0.039) | 0.047(0.170) | 0.060(0.033) | n.s. |
| US_Sn/ug/L | 100 | 0.292±0.282 | 0.213(0.077) | 0.197(0.161) | 0.216(0.061) | n.s. |
| US_Sb/ug/L | 100 | 6.908±2.562 | 6.648(2.987) | 5.114(3.237) | 6.656(2.340) | n.s. |
| US_I/ug/L | 100 | 139.479±436.357 | 55.816(36.607) | 57.571(53.502) | 50.734(32.206) | n.s. |
| US_Tl/ug/L | 53.49 | 0.088±0.231 | 0.015(0.008) | 0.020(0.761) | 0.014(0.007) | n.s. |
| US_Pb/ug/L | 100 | 8.812±5.145 | 7.567(5.184) | 9.127(5.238) | 7.336(3.823) | n.s. |
| US_Fe/mg/L | 100 | 381.84±61.266 | 394.374(34.847) | 403.073(58.926) | 393.563(26.346) | n.s. |

Abbreviations: US, umbilical cord blood of smaller twin; V, vanadium; Cr, chromium; Mn, manganese; Co, cobalt; Ni, nickel; Cu, copper; Zn, zinc; As, arsenic; Se, selenium; Sr, strontium; Mo, molybdenum; Cd, cadmium; Sn, stannum; Sb, stibium; I, iodine; Tl, thallium; Pb, lead; Fe, ferrum

**Table 4**

Differences in the levels of trace elements in umbilical cord blood of both twin

| Elementt/Units | Total  (n=60) | | IBWD≥20% (n=21) | IBWD<20% (n=39) | *P* |
| --- | --- | --- | --- | --- | --- |
|  | Mean±SD | Median | Median | Median |  |
|  |  | (IQR) | (IQR) | (IQR) |  |
| UD_V/ng/L | -0.710±50.530 | 1.466(49.851) | 1.466(38.573) | 1.136(49.851) | n.s. |
| UD_Cr/ug/L | 0.455±3.348 | -0.093(0.538) | -0.068(0.144) | -0.137(0.553) | n.s. |
| UD_Mn/ug/L | -7.126±17.048 | -2.449(11.538) | -4.018(24.782) | -1.957(10.881) | n.s. |
| UD_Co/ug/L | -0.009±0.154 | -0.049(0.139) | -0.051(0.119) | -0.043(0.129) | n.s. |
| UD_Ni/ug/L | -0.416±1.768 | -0.027(0.452) | 0.019(0.548) | -0.055(0.408) | n.s. |
| UD_Cu/ug/L | -41.291±153.689 | -10.869(94.122) | -26.204(135.101) | -8.338(66.000) | n.s. |
| UD_Zn/ug/L | 3.135±246.04 | 1.313(285.699) | 33.837(233.840) | -41.090(246.667) | n.s. |
| UD_As/ug/L | -0.102±0.421 | -0.07(0.303) | -0.084(0.236) | -0.063(0.365) | n.s. |
| UD_Se/ug/L | -1.685±20.103 | -1.226(20.309) | -1.751(17.683) | -0.722(17.508) | n.s. |
| UD_Sr/ug/L | -0.051±2.715 | -0.454(2.93) | 0.105(2.071) | -0.938(2.481) | n.s. |
| UD_Mo/ug/L | 0.025±0.167 | 0.036(0.117) | 0.061(0.166) | 0.031(0.107) | n.s. |
| UD_Cd/ng/L | -1.663±19.827 | 0.000(28.222) | 3.225(25.560) | -4.838(30.641) | n.s. |
| UD_Sn/ng/L | 8.330±254.144 | 7.815(46.890) | 9.618(44.485) | 2.705(45.086) | n.s. |
| UD_Sb/ug/L | 0.033±3.289 | -0.300(2.841) | 0.182(1.674) | -0.426(2.971) | n.s. |
| UD_I/ug/L | -62.522±445.643 | 5.227(49.226) | 3.899(50.815) | 5.319(37.71) | n.s. |
| UD_Tl/ng/L | -0.468±3.791 | 0.132(4.145) | -0.357(4.136) | 0.296(3.574) | n.s. |
| UD_Pb/ug/L | -0.051±3.365 | 0.179(1.750) | 0.363(2.215) | 0.158(1.508) | n.s. |
| UD_Fe/mg/L | 1.072±66.63 | -2.657(41.967) | -5.941(51.099) | -1.109(47.761) | n.s. |

Abbreviations: UD, differences of trace elements in umbilical cord blood of twins (UL-US); V, vanadium; Cr, chromium; Mn, manganese; Co, cobalt; Ni, nickel; Cu, copper; Zn, zinc; As, arsenic; Se, selenium; Sr, strontium; Mo, molybdenum; Cd, cadmium; Sn, stannum; Sb, stibium; I, iodine; Tl, thallium; Pb, lead; Fe, ferrum

**Table 5**

Quantitative levels of trace elements in placenta of larger twin grouped by intertwin birthweight discordance

| Element/Units | Total  (n=60) | | | IBWD≥20%  (n=21) | IBWD<20% (n=39) | *P* |
| --- | --- | --- | --- | --- | --- | --- |
|  | % of detection | Mean±SD | Median  (IQR) | Median  (IQR) | Median  (IQR) |  |
| PL_V/ug/kg | 93.22 | 0.591±0.635 | 0.341(0.715) | 0.846(1.256) | 0.290(0.430) | 0.009 |
| PL_Cr/ug/kg | 100 | 72.416±35.738 | 62.842(42.923) | 53.297(69.889) | 68.554(36.887) | n.s. |
| PL_Mn/ug/kg | 100 | 107.973±36.346 | 100.942(41.320) | 81.714(34.904) | 103.895(42.317) | n.s. |
| PL_Co/ug/kg | 100 | 3.696±2.169 | 3.063(1.774) | 2.540(1.176) | 3.334(2.408) | n.s. |
| PL_Ni/ug/kg | 100 | 17.778±10.182 | 15.041(12.975) | 18.163(13.603) | 14.548(11.272) | n.s. |
| PL_Cu/ug/kg | 100 | 675.379±266.843 | 626.595(227.877) | 615.056(302.157) | 640.679(191.505) | n.s. |
| PL_Zn/ug/kg | 100 | 8590.921±2721.616 | 8174.261(2269.596) | 8463.192(2004.751) | 7879.919(2447.740) | n.s. |
| PL_As/ug/kg | 96.61 | 0.835±0.437 | 0.775(0.602) | 0.716(0.592) | 0.806(0.593) | n.s. |
| PL_Se/ug/kg | 100 | 94.120±24.979 | 92.889(26.242) | 89.52(38.626) | 94.086(29.317) | n.s. |
| PL_Sr/ug/kg | 100 | 203.153±322.026 | 92.569(127.169) | 72.681(57.112) | 108.566(125.259) | 0.009 |
| PL_Mo/ug/kg | 100 | 12.888±8.006 | 10.402(6.110) | 11.205(12.719) | 10.402(5.208) | n.s. |
| PL_Cd/ug/kg | 100 | 9.761±5.885 | 8.310(8.772) | 7.423(4.642) | 9.328(10.795) | n.s. |
| PL_Sn/ug/kg | 100 | 6.024±3.851 | 4.630(5.036) | 7.281(7.248) | 4.180(3.481) | n.s. |
| PL_Sb/ug/kg | 93.22 | 0.471±0.771 | 0.305(0.35) | 0.483(0.556) | 0.246(0.229) | n.s. |
| PL_I/ug/kg | 100 | 115.897±216.627 | 54.227(93.957) | 80.449(72.726) | 35.491(74.444) | 0.013 |
| PL_Tl/ug/kg | 100 | 0.128±0.091 | 0.104(0.136) | 0.104(0.136) | 0 |  |
| PL_Pb/ug/kg | 100 | 11.989±14.802 | 8.926(5.829) | 8.823(4.857) | 8.926(7.877) | n.s. |
| PL_Fe/mg/kg | 100 | 41.760±14.431 | 40.757(21.397) | 38.282(24.695) | 40.757(19.203) | n.s. |

Abbreviations: PL, placenta of larger twin; V, vanadium; Cr, chromium; Mn, manganese; Co, cobalt; Ni, nickel; Cu, copper; Zn, zinc; As, arsenic; Se, selenium; Sr, strontium; Mo, molybdenum; Cd, cadmium; Sn, stannum; Sb, stibium; I, iodine; Tl, thallium; Pb, lead; Fe, ferrum

**Table 6**

Quantitative levels of trace elements in placenta of smaller twin

| Element/Units | Total  (n=60) | | | IBWD≥20% (n=21) | IBWD<20%  (n=39) | *P* |
| --- | --- | --- | --- | --- | --- | --- |
|  | % of detection | Mean±SD | Median | Median | Median |  |
|  |  |  | (IQR) | (IQR) | (IQR) |  |
| PS_V/ug/kg | 94.83 | 0.580±0.954 | 0.346(0.287) | 0.414(0.879) | 0.301(0.305) | n.s. |
| PS_Cr/ug/kg | 100 | 89.001±88.532 | 60.952(48.311) | 52.958(29.364) | 69.9(72.654) | n.s. |
| PS_Mn/ug/kg | 100 | 121.914±61.155 | 107.373(58.331) | 100.191(58.331) | 110.658(62.495) | n.s. |
| PS_Co/ug/kg | 100 | 3.728±2.189 | 3.262(2.078) | 2.885(1.652) | 3.374(2.642) | n.s. |
| PS_Ni/ug/kg | 100 | 22.339±18.514 | 17.773(16.608) | 18.289(17.619) | 17.615(17.511) | n.s. |
| PS_Cu/ug/kg | 100 | 760.289±353.976 | 644.224(417.762) | 655.209(717.773) | 633.239(380.935) | n.s. |
| PS_Zn/ug/kg | 100 | 8292.301±3458.857 | 7529.457(2239.344) | 7556.000(2225.647) | 7368.148(2201.152) | n.s. |
| PS_As/ug/kg | 100 | 0.832±0.519 | 0.699(0.671) | 0.662(0.768) | 0.804(0.620) | n.s. |
| PS_Se/ug/kg | 100 | 96.737±25.625 | 94.309(35.934) | 90.326(38.616) | 99.654(36.34) | n.s. |
| PS_Sr/ug/kg | 100 | 275.610±454.445 | 125.164(177.514) | 74.867(197.672) | 127.445(206.102) | n.s. |
| PS_Mo/ug/kg | 100 | 11.603±5.119 | 10.652(6.019) | 8.783(4.932) | 11.036(7.254) | 0.001 |
| PS_Cd/ug/kg | 100 | 10.681±7.730 | 9.088(8.441) | 6.462(5.189) | 11.082(13.086) | n.s. |
| PS_Sn/ug/kg | 100 | 7.691±10.931 | 5.267(3.232) | 5.302(8.164) | 5.174(3.702) | n.s. |
| PS_Sb/ug/kg | 98.28 | 0.555±0.918 | 0.322(0.420) | 0.424(0.578) | 0.321(0.296) | n.s. |
| PS_I/ug/kg | 100 | 118.986±257.056 | 32.564(51.136) | 40.767(142.402) | 29.659(50.354) | n.s. |
| PS_Tl/ug/kg | 100 | 0.223±0.153 | 0.205(0.225) | 0.205(0.225) | 0 |  |
| PS_Pb/ug/kg | 100 | 12.937±8.437 | 10.611(8.022) | 8.093(4.153) | 12.162(9.104) | 0.020 |
| PS_Fe/mg/kg | 100 | 40.325±15.866 | 37.284(18.783) | 35.256(18.047) | 39.246(24.310) | n.s. |

Abbreviations: PS, placenta of smaller twin; V, vanadium; Cr, chromium; Mn, manganese; Co, cobalt; Ni, nickel; Cu, copper; Zn, zinc; As, arsenic; Se, selenium; Sr, strontium; Mo, molybdenum; Cd, cadmium; Sn, stannum; Sb, stibium; I, iodine; Tl, thallium; Pb, lead; Fe, ferrum

**Table 7**

Differences in the levels of trace elements in placenta of both twin

| Element/Units | Total  (n=60) | | IBWD≥20%  (n=21) | IBWD<20% (n=39) | *P* |
| --- | --- | --- | --- | --- | --- |
|  | Mean±SD | Median | Median | Median |  |
|  |  | (IQR) | (IQR) | (IQR) |  |
| PD_V/ug/kg | 0.002±1.095 | -0.002(0.596) | 0.059(0.753) | -0.043(0.491) | n.s. |
| PD_Cr/ug/kg | -17.361±91.901 | -1.696(44.408) | 6.595(31.964) | -6.212(66.178) | n.s. |
| PD_Mn/ug/kg | -13.756±59.976 | -1.582(44.210) | -16.721(47.099) | 3.443(45.893) | n.s. |
| PD_Co/ug/kg | -0.026±2.337 | -0.027(2.028) | 0.221(1.975) | -0.051(2.009) | n.s. |
| PD_Ni/ug/kg | -4.856±19.171 | -2.200(14.587) | -0.297(9.219) | -2.885(16.476) | n.s. |
| PD_Cu/ug/kg | -84.115±346.282 | -24.664(277.988) | -51.692(457.776) | -20.44(282.879) | n.s. |
| PD_Zn/ug/kg | 34.495±3816.044 | 385.061(2454.543) | 1353.433(3082.967) | 220.773(2380.187) | n.s. |
| PD_As/ug/kg | 0.009±0.441 | 0.050(0.530) | 0.091(0.563) | -0.089(0.545) | n.s. |
| PD_Se/ug/kg | -2.743±26.709 | -0.172(25.883) | -3.344(21.456) | 2.846(38.384) | n.s. |
| PD_Sr/ug/kg | -69.870±433.119 | -19.672(209.559) | -25.661(221.731) | -5.793(171.988) | n.s. |
| PD_Mo/ug/kg | 1.044±8.260 | 0.317(5.771) | 1.149(7.016) | -0.440(6.512) | 0.013 |
| PD_Cd/ug/kg | -0.862±4.137 | -0.096(3.807) | -0.032(2.865) | -0.109(5.102) | n.s. |
| PD_Sn/ug/kg | -1.668±11.020 | -0.510(4.638) | -0.521(4.841) | -0.404(4.797) | n.s. |
| PD_Sb/ug/kg | -0.083±1.205 | -0.013(0.415) | 0.104(0.469) | -0.068(0.360) | n.s. |
| PD_I/ug/kg | -3.425±209.374 | 8.465(55.945) | 20.375(128.624) | 5.832(46.604) | n.s. |
| PD_Tl/ug/kg | -0.095±0.065 | -0.101(0.089) | -0.101(0.089) | 0 |  |
| PD_Pb/ug/kg | -0.994±16.210 | -1.36(6.062) | -0.282(4.877) | -2.456(10.63) | n.s. |
| PD_Fe/mg/kg | 1.347±18.861 | 1.666(19.058) | 0.602(13.08) | 2.663(20.368) | n.s. |

Abbreviations: PD, differences of trace elements in placenta of twins (PL-PS); V, vanadium; Cr, chromium; Mn, manganese; Co, cobalt; Ni, nickel; Cu, copper; Zn, zinc; As, arsenic; Se, selenium; Sr, strontium; Mo, molybdenum; Cd, cadmium; Sn, stannum; Sb, stibium; I, iodine; Tl, thallium; Pb, lead; Fe, ferrum
